# Attention-Enhanced CNN-LSTM Model for Exercise Oxygen Consumption Prediction with Multi-Source Temporal Features

**DOI:** 10.3390/s25134062

**Published:** 2025-06-29

**Authors:** Zhen Wang, Yingzhe Song, Lei Pang, Shanjun Li, Gang Sun

**Affiliations:** Institute of Artificial Intelligence in Sports, Capital University of Physical Education and Sports, Beijing 100191, China; wangzhen23@cupes.edu.cn (Z.W.); songyingzhe2022@cupes.edu.cn (Y.S.); sungang@cupes.edu.cn (G.S.)

**Keywords:** oxygen uptake, deep learning, neural network, attention mechanism

## Abstract

Dynamic oxygen uptake (VO_2_) reflects moment-to-moment changes in oxygen consumption during exercise and underpins training design, performance enhancement, and clinical decision-making. We tackled two key obstacles—the limited fusion of heterogeneous sensor data and inadequate modeling of long-range temporal patterns—by integrating wearable accelerometer and heart-rate streams with a convolutional neural network–LSTM (CNN-LSTM) architecture and optional attention modules. Physiological signals and VO_2_ were recorded from 21 adults through resting assessment and cardiopulmonary exercise testing. The results showed that pairing accelerometer with heart-rate inputs improves prediction compared with considering the heart rate alone. The baseline CNN-LSTM reached *R*^2^ = 0.946, outperforming a plain LSTM (*R*^2^ = 0.926) thanks to stronger local spatio-temporal feature extraction. Introducing a spatial attention mechanism raised accuracy further (*R*^2^ = 0.962), whereas temporal attention reduced it (*R*^2^ = 0.930), indicating that attention success depends on how well the attended features align with exercise dynamics. Stacking both attentions (spatio-temporal) yielded *R*^2^ = 0.960, slightly below the value for spatial attention alone, implying that added complexity does not guarantee better performance. Across all models, prediction errors grew during high-intensity bouts, highlighting a bottleneck in capturing non-linear physiological responses under heavy load. These findings inform architecture selection for wearable metabolic monitoring and clarify when attention mechanisms add value.

## 1. Introduction

Cardiorespiratory fitness is an important indicator of all-cause mortality risk [1] and also plays a key role in endurance performance [2]. Oxygen consumption (VO_2_) and its dynamic response during exercise are widely used in the assessment of cardiorespiratory fitness. The analysis of VO_2_ during exercise provides important physiological information about the components of the aerobic metabolism system, including the cardiopulmonary and muscular systems [3]. Furthermore, abnormal oxygen consumption responses during exercise may precede clinical manifestations of disease, thereby demonstrating significant practical value in disease warning and exercise risk screening [4].

Traditional oxygen consumption monitoring relies on laboratory metabolic chambers (such as the TrueOne 2400, ParvoMedic Inc., Salt Lake City, UT, USA), which can obtain energy metabolism data at rest and during exercise through high-precision gas analysis systems. However, their operation is strictly limited to laboratory environments, and the equipment is bulky and expensive, making it difficult to meet the dynamic monitoring requirements of sports venues [5]. Portable oxygen consumption monitoring devices currently available on the market (such as K5, Cosmed S.r.l., Rome, Italy and VO_2_ Master, VO_2_ Master Health Sensors Inc., Vernon, BC, Canada) have broken through the limitations of laboratory environments and enabled the on-site detection of respiratory data during exercise. However, during testing, factors such as wearing respiratory masks that alter the natural breathing pattern of participants, frequent gas calibration procedures, and the high cost of equipment purchase resulted in a certain degree of error between the actual measured oxygen uptake data of exercisers and their true physiological values [6].

The development of wearable sensor technology has provided new ideas for non-invasive oxygen consumption monitoring. Early studies primarily used the heart rate (HR) as an assessment indicator and employed traditional statistical models such as linear regression [7] to estimate oxygen uptake at each moment during exercise [8]. However, the above studies did not fully explore the dynamic information contained in the evolution of heart rate sequences over time. In recent years, artificial intelligence technologies represented by deep learning have opened up more development opportunities for real-time, dynamic oxygen consumption calculations. Deep learning algorithms can more deeply explore the relationship between other physiological signals and oxygen consumption, making oxygen consumption monitoring during exercise more convenient and accurate. Therefore, recent studies have begun to focus on utilizing the time-dependent nature of oxygen uptake during exercise and its correlation with multiple physiological indicators, combined with more complex deep learning prediction models to predict real-time oxygen uptake responses during exercise [9].

A multi-indicator model refers to an oxygen consumption prediction model that uses multiple relevant factors from different data sources as inputs. Typical input indicators include static characteristics (e.g., age, BMI, etc.) and dynamic movement characteristics (e.g., heart rate, acceleration, etc.). They are associated with oxygen consumption by reflecting metabolic basis and exercise intensity. When performing in-depth information mining on the above multi-dimensional features, convolutional neural networks (CNNs) are commonly used because they can extract potential features from adjacent input indicators as spatial features in deep learning models [10]. However, there is no spatial adjacency relationship between the characteristics during the movement process similar to image pixels, and the order of the indicators does not have a clear spatial structure. Therefore, although local convolutions using CNNs can extract some potential features, it is still difficult to comprehensively capture the complex potential relationships between multiple indicators.

A time series refers to the temporal correlation between monitored data sequences. Indicators such as the heart rate, acceleration, and oxygen consumption during exercise tend to change with the duration of exercise, and past data sequences are correlated with future data sequences. Long Short-Term Memory (LSTM) networks are capable of remembering time series information. However, as the time series in the data lengthens, LSTM may not remember early data points well enough [11], requiring further improvement to strengthen temporal modeling.

**Figure 1:**
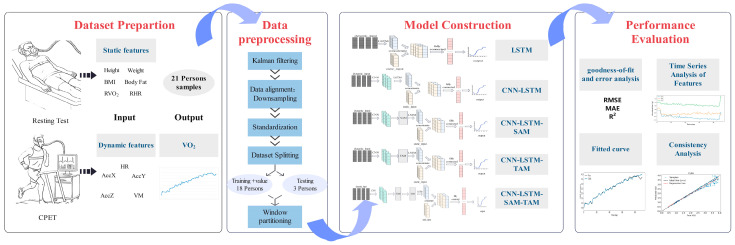
Attention-CNN-LSTM framework for VO_2_ prediction using multi-source temporal features.

In summary, existing VO_2_ prediction studies still have shortcomings in the deep integration of multi-indicator information, the modeling of long-term dependencies in temporal features, and the dynamic extraction of key features. This paper proposes an oxygen consumption prediction model based on the CNN-LSTM structure and incorporates spatial and temporal attention mechanisms into the model to enhance prediction performance. As shown in Figure 1, the main tasks are as follows: (1) conducting resting experiments and cardiopulmonary exercise tests (CPETs) to collect physiological data, (2) constructing LSTM and CNN-LSTM dynamic oxygen consumption prediction models based on input features derived from the integration of static and dynamic indicators, and, (3) based on the CNN-LSTM model, adding a time, space, and spatio-temporal attention mechanism to construct an oxygen consumption prediction model and conduct a comparative analysis.

## 2. Materials and Methods

### 2.1. Participants

This study recruited 21 participants, including 14 males and 7 females, aged between 21 and 28 years. Table 1 shows the demographic characteristics of the subjects. This study was approved by the Institutional Review Board, and all participants signed written informed consent forms. Each participant underwent a health assessment screening to assess potential risks, had had no hospitalization records in the past six months, and was able to complete the Physical Activity Readiness Questionnaire (PAR-Q) for the physical activity programmed. We excluded participants with medical implantable electronic devices, those who had sustained running injuries, or those at high risk of injury.

### 2.2. Experimental Design and Data Collection

The data sources included two parts: resting test and CPET. Before the resting test, subjects were asked to fast for at least 4 h and refrain from consuming caffeinated beverages or alcohol for 24 h. They were also asked to avoid strenuous exercise for 48 h before the test to ensure that they were in a resting state before the test. Each subject was asked to close their eyes and lie still for 15 min before the test began to ensure that they were completely relaxed. After the resting testing began, the subjects continued to lie quietly with their eyes closed and wore a heart rate belt (H10, Polar Electro Oy, Kempele, Finland) with a sampling frequency of 1 Hz. At the same time, a gas metabolism analyzer (Powercube-Ergo, Ganshorn, Niederlauer, Germany) was used to conduct a 10-min resting oxygen uptake test, with a sampling frequency of 0.1 Hz. The device recorded their breathing data and heart rates.

Before the CPET, the subjects first did a 10-min slow jog to warm up. After entering the formal testing phase, participants used a treadmill to perform incremental exercise according to the Ramp protocol [12], with the treadmill speed increasing by 1 km/h per minute and the incline remaining at 0%. During the test, accelerometers (WT901BLECL5.0, Witmotion, Shenzhen, China) were worn on the wrists of the subjects’ non-dominant hands to collect acceleration data at a frequency of 10 Hz. Heart rate belts collected heart rate data and gas metabolism analyzers collected and recorded oxygen consumption during exercise. The test was terminated when the subject met any two of the following criteria: heart rate reached 90% of maximum heart rate; Respiratory Quotient > 1.15; Rating of Perceived Exertion > 17; oxygen uptake plateaued. The maximum heart rate was calculated using the following equation: HRmax = 208 − 0.7 × age.

### 2.3. Data Preprocessing

For multi-source heterogeneous data such as heart rate, acceleration, and gas parameters during exercise, time synchronization was first performed based on the experimental records before preprocessing. To suppress random noise and obtain smooth one-dimensional acceleration estimates, this study applied a Kalman filter to the original acceleration signals. The following example uses the original acceleration sequence (*a_x_*) on the X-axis to illustrate the implementation details of the Kalman filter. The remaining Y- and Z-axis signals use the same model and hyperparameters and are processed independently in parallel. The sampling frequency of the original acceleration data is 10 Hz (Δt=0.1 s). The filter uses a one-dimensional random walk assumption, and its state-observation model is $x_{k}\;=x_{k-1}\;+w_{k-1},z_{k}\;=x_{k}\;+v_{k}$. Here, $x_{k}$ denotes the ‘true’ X-axis acceleration (in units of g) of frame *k*, $z_{k}$ denotes the corresponding raw measurement value, $w_{k-1}∼N\left(0,Q\right)$, and $v_{k}∼N\left(0,R\right)$. Based on the stationary segment noise calibration and Allan variance analysis, the parameters are set as A=H=1, $Q=0.01g^{2}$, and $R=0.10g^{2}$. The initial state estimate is set as the first frame measurement $x_{0}=a_{x}\left(0\right)$, and the initial covariance $P_{0}=1g^{2}$. After that, the three directional accelerations are combined into a scalar composite value, *VM*.

(1) $$VM=\sqrt{AccX^{2}+AccY^{2}+AccZ^{2}}$$

To address the issue of accelerometer sampling frequency being higher than heart rate belt frequency, a down sampling method was used to align the accelerometer data with the heart rate data. After that, the features were divided into dynamic features and static features (Table 2) and standardized using Z-scores (Equation (2)).

(2) $$X^{\prime }=\frac{X-\mu }{\sigma }$$

*μ* represents the mean of all sample data and *σ* represents the standard deviation of all sample data.

**Table 2:** Classification of dynamic and static characteristics.

Feature Category	Feature
Static Features	Weight (kg)
	Height (m)
	BMI (kg/m^2^)
	Body fat percentage (%)
	Resting oxygen consumption (L/min)
	Resting heart rate (Beats/min)
Dynamic Features	Exercise heart rate (Beats/min)
	X-axis acceleration (G)
	Y-axis acceleration (G)
	Z-axis acceleration (G)
	VM (G)

To coordinate the sampling frequency of the gas analyzer with other devices, a 10-s non-overlapping time window was constructed, and the dynamic characteristics within the window were serialized in 1-s increments. The absolute oxygen consumption values collected by the gas analyzer were used as label values for each window of the model. The missing acceleration and heart rate values in the window were filled in using the average values in this window.

### 2.4. Model Construction

#### 2.4.1. Attention Mechanism

Attention mechanisms (AMs) are often used to solve temporal and spatial problems encountered in modeling. By dynamically allocating weights to different indicators or time steps, AMs can highlight key information based on their correlations, thereby assisting predictive models in more accurately capturing key features [13]. This study adopted three attention mechanisms to optimize the spatio-temporal features of multi-source heterogeneous data for oxygen consumption prediction: Spatial Attention Module (SAM), Temporal Attention Module (TAM), and Spatio-temporal Attention Module (STAM). SAM can extract potential features from multiple input variables and analyze their importance to the predicted target indicator. This solves the limitation of CNNs in feature modeling of adjacent input indicators. TAM focuses on the most critical part of time sequence in accurate time prediction. It assigns corresponding weights, reducing the time information that LSTM needs to remember [14].

(1) Time Attention Mechanism (TAM)

**Figure 2:**
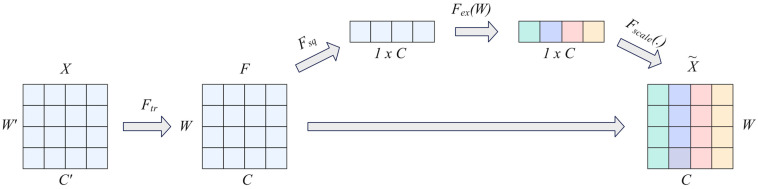
Squeeze-Excitation (SE) module.

The Squeeze-Excitation (SE) module is a temporal attention mechanism that enhances the representational capacity of convolutional neural networks through dynamic channel feature re-labelling [15]. The actual implementation process is shown in Figure 2. In the figure, *X* represents the input data, *C′* and *C* represent time series, *W′* and *W* represent spatial dimensions (multiple indicators), *F* represents feature maps, *F_tr_* represents convolution, *F_sq_* represents feature map compression, *F_ex_* represents feature map excitation, and *Fscale* represents feature re-calibration.

The core idea of this mechanism lies in explicitly modeling the non-linear interaction between channel dimensions. Specifically, it consists of two stages:

- Squeeze: Spatial features (channel number *C* and two-dimensional spatial dimensions *H* × *W*) are compressed along the spatial dimension into channel description vectors through global average pooling. Compared with the *C × H × W* structure of image data, this paper omits the spatial dimension *H × W* of the time series and retains only the time channel *C* and the spatial dimension *W* composed of multiple indicators. The calculation method is shown in Equation (3). *F_C_* denotes the feature matrix *F* on the *C*th channel and *F_C_*(*i*) denotes its value at the *i*th time step.
  (3) $$F_{C}\;=F[:,C]\in R^{W},\;Z_{C}=\frac{1}{W}\sum_{i=1}^{W}F_{C}(i)$$
- Excitation: We introduce a fully connected layer with a bottleneck structure to generate channel attention weights *S*:
  (4) $$S=\sigma (W_{2}\cdot \delta (W_{1}\cdot Z))$$

$W_{1}\in R^{\frac{C}{r}\times C}$ and $W_{2}\in R^{C\times \frac{C}{r}}$ are learnable parameters (*r* is the dimension reduction ratio), *δ* is the Relu activation function, and *σ* is the Sigmoid gate function.

Finally, the original features are re-calibrated using channel-wise weight *S_C_*.

(5) $$F_{C}^{\prime }=S_{C}\cdot F_{C}$$

(2) Spatial Attention Mechanism (SAM)

According to the spatial attention mechanism mentioned by Woo in their study [16], we define ‘spatial attention’ as modeling the importance of feature dimensions at different positions in a time series to characterize ‘which feature dimensions should be focused on at different time steps’.

**Figure 3:**
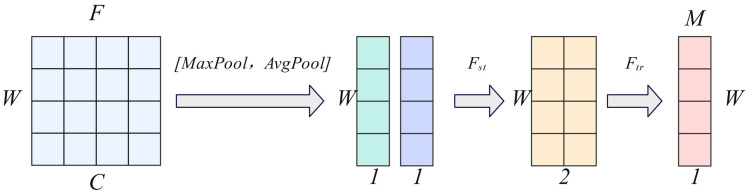
Spatial attention mechanism.

The SAM implementation process is shown in Figure 3, where *C* represents the time series, *W* represents the spatial dimension (multiple indicators), *F* represents the feature map, *F_st_* represents feature concatenation, *F_tr_* represents convolution, and *M* represents the spatial attention map. First, we perform maximum-pooling and average-pooling operations on the input features *F* along the time dimension *C* to obtain two one-dimensional representations: $F_{avg}^{S},F_{max}^{S}\in R^{1\times W}$. These two representations reflect the average response intensity and strongest response across all time steps for each feature dimension, thereby comprehensively modeling the importance of each dimension. Subsequently, we concatenate the two in the channel dimension to form a 2 × *W* fusion feature representation, which is then inputted into a one-dimensional convolutional layer to extract local structural information and generate attention weights. Finally, the output is normalized using the Sigmoid function to obtain the spatial attention map $M_{S}\in R^{1\times W}$, which is then multiplied element-wise with the original input features (*F*) to achieve weighted adjustment in feature dimension. The calculation equations are shown in Equations (6) and (7).

(6) $$M_{S}(F)=\sigma (f([AvgPool(F);MaxPool(F)]))=\sigma (f([F_{avg}^{S};F_{max}^{S}]))$$

(7) $$F\prime =M_{S}(F)⊗F$$

(3) Spatio-temporal Attention Mechanism (STAM)

**Figure 4:**
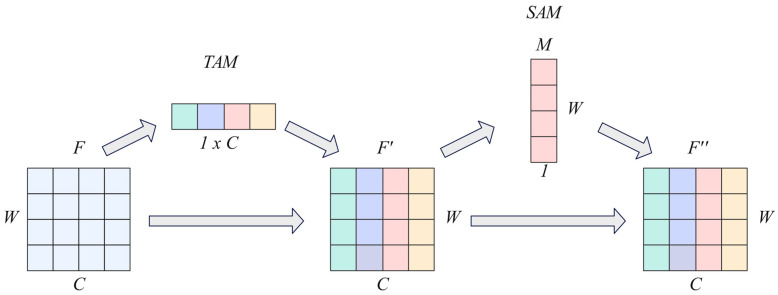
Spatio-temporal Attention Mechanism.

The Spatio-temporal Attention Mechanism consists of a TAM and a SAM (Figure 4), which can jointly model the temporal dynamics of time series and the multi-indicator spatial correlation. This mechanism adopts a cascading structure: input features first pass through TAM, which aggregates information along the indicator dimension to generate temporal step importance weights, highlighting key temporal segments. Subsequently, pooling is performed along the time axis and the dependencies between multiple indicators are learned, and indicator weights are generated through convolution. Finally, the temporal and spatial attention weights are applied to the input features in stages to refine the features in both the temporal and metric dimensions.

#### 2.4.2. VO_2_ Prediction Model

**Figure 5:**
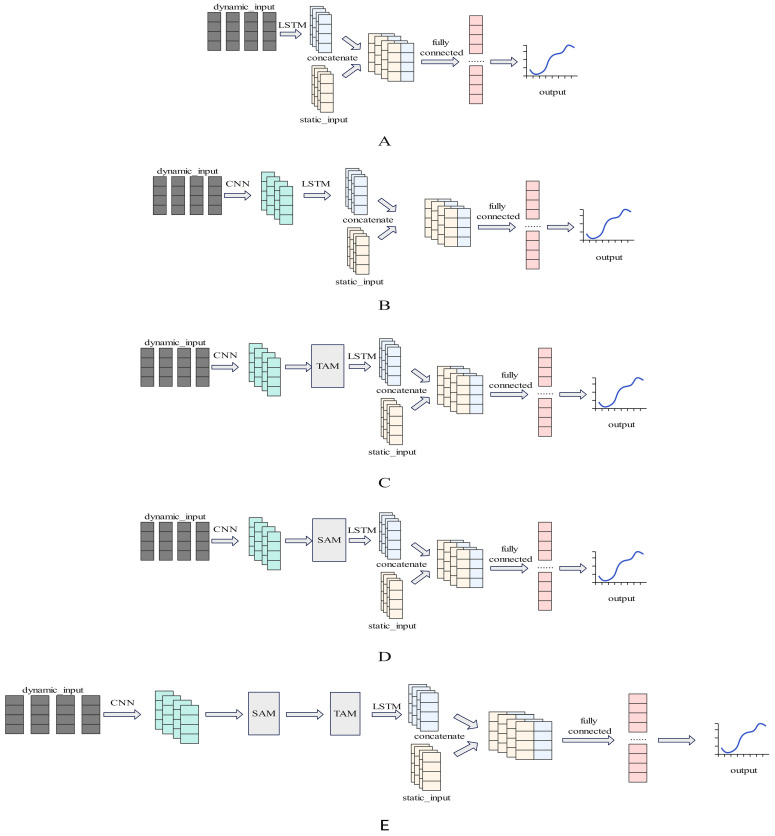
Structure of oxygen uptake prediction models: (**A**)—LSTM; (**B**)—CNN-LSTM; (**C**)—CLTA; (**D**)—CLSA; (**E**)—CLSTA.

This paper proposes a deep learning model for dynamic oxygen uptake prediction. First, to validate the effectiveness of CNNs for multi-indicator fusion, an independent LSTM model (Figure 5A) was constructed, which directly receives raw time-series inputs and ignores spatial feature extraction across indicators. Second, we constructed a CNN-LSTM model and used it as the baseline model for this study (Figure 5B). It extracts spatial features between multiple indicators through convolution operations and captures time dependency using LSTM.

In order to improve the model’s sensitivity to key features, we introduced the three attention mechanisms described in Section 2.4.1 to the baseline model. The CNN-TAM-LSTM (CLTA) model embeds TAM before the LSTM layer (Figure 5C), aggregates the mean and maximum values along the indicator dimension, generates time step weights, and enhances the feature responses of key time periods. The CNN-SAM-LSTM model (CLSA) embeds SAM before the LSTM layer (Figure 5D) to learn indicator importance weights through time dimension pooling. CNN-SAM-TAM-LSTM (CLSTA) cascades SAM and TAM (Figure 5E) achieve joint optimization of temporal sensitivity and indicator correlation.

The model inputs six static features and five dynamic features (Table 2) separately and predicts the oxygen uptake at the current time step as the output. Using the Adam optimizer, the learning rate is set to 0.001 and the batch size is set to 32. We divide the data of all 21 people into two groups: 3 people as an independent test set and the remaining 18 people for six-fold cross-validation. In each round of division, we divide the 18 people into 6 groups, take 1 group as the validation set in turn, and use the remaining 5 groups as the training set. The specific parameters of each layer of the model are shown in Table 3.

### 2.5. Model Evaluation Indicators

In order to comprehensively quantify the accuracy, stability, and time alignment capability of the dynamic oxygen uptake prediction model, this study comprehensively selected evaluation indicators.

(1) Root Mean Square Error (RMSE)
  (8) $$RMSE=\sqrt{\frac{1}{N}\sum_{i=1}^{N}{\left(y_{i}-{\hat{y}}_{i}\right)}^{2}}$$

We measure the average deviation between the predicted value and the actual value. *N* is the total number of samples; $y_{i}$ and ${\hat{y}}_{i}$ are the true value and predicted value of the *i*-th sample, respectively.

(2) Mean Absolute Error (MAE)
  (9) $$MAE=\frac{1}{N}\sum_{i=1}^{N}\left|y_{i}-{\hat{y}}_{i}\right|$$

We calculate the absolute average value of the prediction error. The symbols have the same meanings as in the RMSE equation.

(3) Deciding Coefficient (*R*^2^)
  (10) $$R^{2}=1-\frac{\sum_{i=1}^{N}{\left(y_{i}-{\hat{y}}_{i}\right)}^{2}}{\sum_{i=1}^{N}{\left(y_{i}-{\bar{y}}_{i}\right)}^{2}},\bar{y}=\frac{1}{N}\sum_{i=1}^{N}y_{i}$$
  We evaluate the explanatory power of the model for changes in oxygen uptake, ranging from [0, 1], where values closer to 1 indicate a higher degree of model fit. $\bar{y}$ represents the arithmetic mean of the actual oxygen uptake values, and the meanings of the other symbols are the same as in the RMSE equation.

## 3. Results

### 3.1. Sequential Dynamic Characteristics

**Figure 6:**
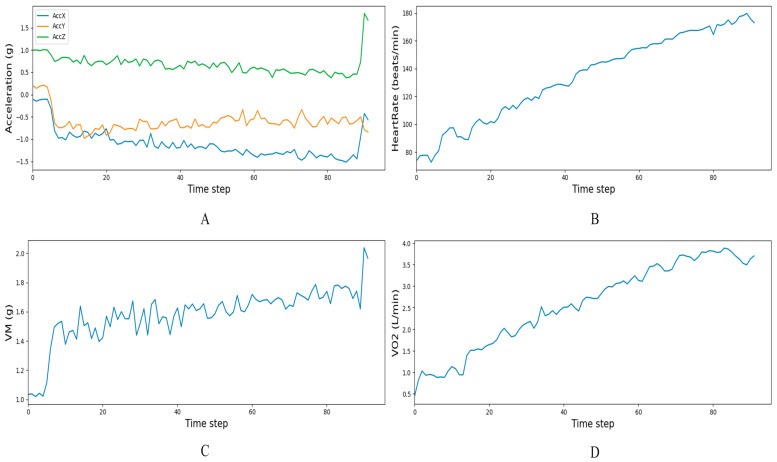
Time dynamics of three-axis acceleration, heart rate, VM, and oxygen uptake during incremental exercise: (**A**)—three-axis acceleration, (**B**)—heart rate, (**C**)—VM, and (**D**)—VO_2_.

We plotted the time-series changes in the dynamic indicators (including triaxial acceleration, heart rate, VM, and oxygen uptake) of a subject during exercise with increasing load, as shown in Figure 6. The dynamic response patterns of the physiological indicators of this subject were consistent with the group data in this study and can be used as typical examples to intuitively illustrate common patterns. The three-axis acceleration signals (Figure 6A) showed obvious fluctuations at the beginning of the movement due to the insufficient coordination of movements. After entering the stable running phase, the acceleration of each axis showed rhythmic fluctuations around the average value due to the regular alternation of steps. Finally, during the sprinting phase, the violent kicking movements and large trunk swings together led to a significant increase in the intensity of the fluctuations. The heart rate (Figure 6B) increased gradually from the resting value with the increase in random exercise intensity and remained generally upward throughout the exercise, approaching the maximum heart rate at the end. The amplitude of VM (Figure 6C) increased continuously with increasing movement intensity. Due to vector synthesis, single-axis-specific noise was suppressed, and the local variance was significantly lower than that of single-axis data. VO_2_ rose slowly in the initial stage and eventually reached a plateau as intensity continued to increase, tending toward the individual’s maximum oxygen uptake (Figure 6D).

### 3.2. Construction of VO_2_ Prediction Model Based on Dynamic-Static Feature Fusion

In developing the oxygen uptake prediction model, an LSTM network was initially constructed to process time-series data. In order to compare and analyze the impact of integrating different dynamic indicators with static indicators on the performance of the prediction model, static features plus heart rate and static features plus acceleration data and the heart rate were used as model inputs. In Table 4, the RMSE, MAE, and *R*^2^ values for the training set, validation set, and test set after six-fold cross-validation are given. The results indicate that relying solely on heart rate signals to predict VO_2_ during exercise is less effective overall than models that combine heart rate and acceleration signals. Among these, after adding the acceleration signal, the RMSE of the LSTM model on the test set decreased from 0.3335 to 0.2317, and *R*^2^ increased from 0.8882 to 0.9460, indicating that the model could more accurately and reliably characterize changes in VO_2_. This result was consistent with the dynamic characteristic analysis in Section 3.1. It was precisely because acceleration could capture short-term violent movements and other intensity fluctuations that it compensated for the delay in heart rate response to VO_2_ changes, thereby significantly improving the estimation accuracy of energy expenditure and oxygen consumption.

A CNN can extract deep features more effectively, so we further compared the performance of the LSTM and CNN-LSTM models in dynamic VO_2_ prediction. The results showed that all models performed better on the training set than on the test set. The models minimized the loss function during the training phase while the test phase measured their generalization ability on unseen data. Therefore, a certain degree of performance degradation was normal. The hybrid model with a CNN layer (CNN-LSTM) outperformed the pure LSTM model in VO_2_ prediction accuracy. When using heart rate and static characteristics as input variables, the CNN-LSTM model achieved an RMSE of 0.3232 on the test set, which was better than the corresponding LSTM model’s 0.3335; *R*^2^ was improved from 0.8882 for the LSTM model to 0.8950. After adding acceleration data to the input variables, the CNN-LSTM model achieved an RMSE of 0.2317 on the test set, outperforming the corresponding LSTM model’s 0.2720; the *R*^2^ value improved from 0.9256 for the LSTM model to 0.9460. This indicates that the introduction of the convolutional structure effectively enhanced the model’s ability to capture real VO_2_ change trends.

### 3.3. VO_2_ Prediction Model with Integrated Attention Mechanism

Attention mechanisms are generally believed to enable models to learn to ‘focus on key points’ when processing information, like humans do, thereby improving their ability to model complex data and their interpretability. This section proposes a dynamic VO_2_ prediction model based on the fusion of time, space, and spatio-temporal attention in the CNN-LSTM model. Since the combination of heart rate and acceleration data with static characteristics is beneficial to model performance, this combination will be used as input in subsequent analyses. The results are shown in Table 5. Compared with the original CNN-LSTM model without the attention mechanism in Section 3.2, the performance of the model was significantly improved after introducing the Spatial Attention Module (CLSA). On both the validation set and the test set, the CLSA model achieved lower RMSE and MAE values and higher *R*^2^ values (the *R*^2^ value on the test set increased from 0.9460 to 0.9621) compared to the best-performing CNN-LSTM model in Section 3.2. In contrast, the CLTA model, which only introduced time attention, did not bring any performance gains. The error on the test set was even slightly higher than that of the original model (RMSE = 0.2648, MAE = 0.1881, *R*^2^ = 0.9295).

At the same time, the CLSTA model, which combined temporal and spatial attention, achieved extremely high goodness of fit on the training set (training set *R*^2^ = 1.000), but its performance in the validation and testing phases (testing set *R*^2^ = 0.9586) was not optimal (as shown in Figure 6), being slightly inferior to the CLSA model containing only spatial attention (testing set *R*^2^ = 0.9621).

### 3.4. VO_2_ Prediction Performance Across Exercise Intensity Zones

**Figure 7:**
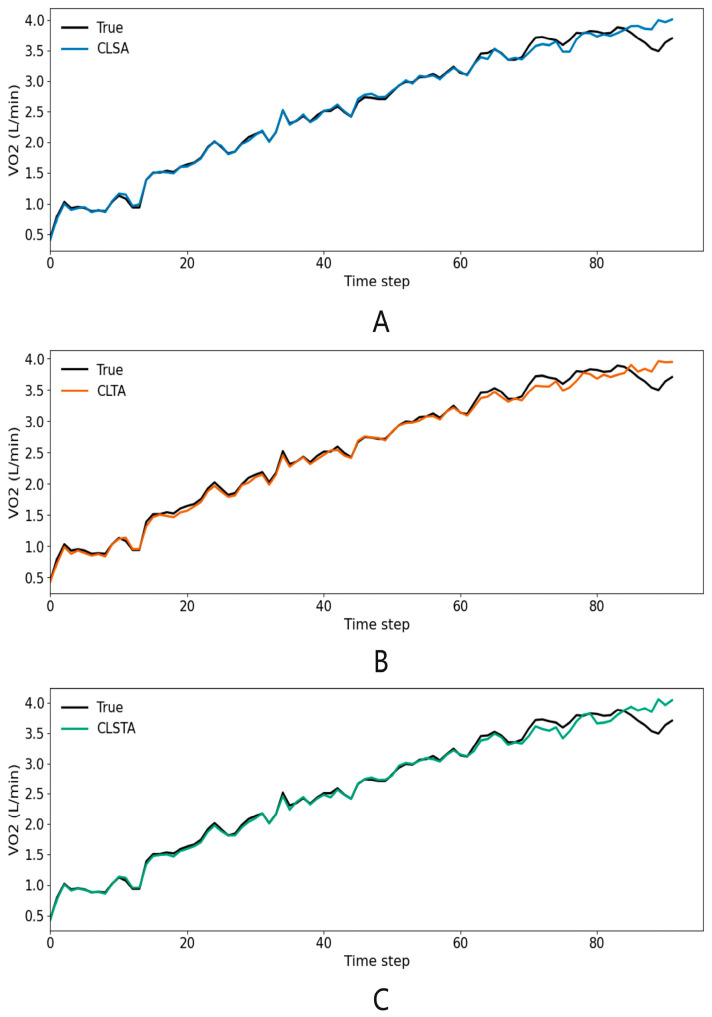
Predicted vs. actual VO_2_ curves during exercise from different models: (**A**) CLSA, (**B**) CLTA, and (**C**) CLSTA.

**Figure 8:**
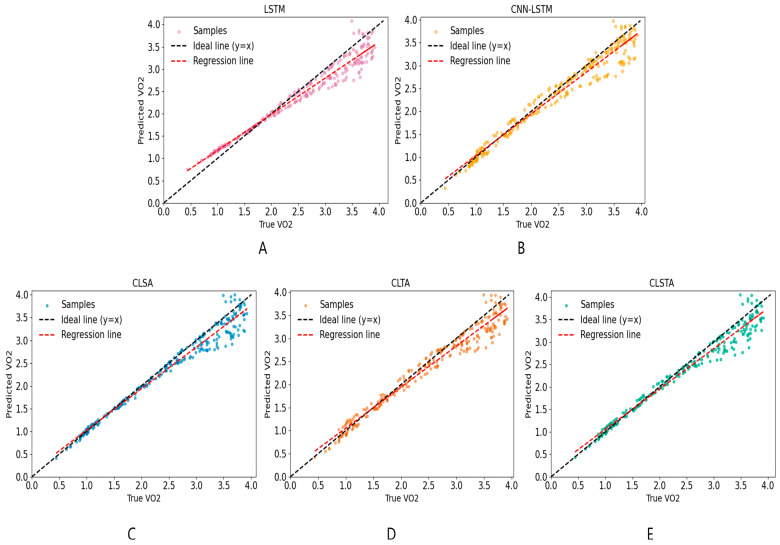
Predicted vs. true VO_2_ scatter-regression plots on the test set for five models: (**A**)—LSTM, (**B**)—CNN-LSTM, (**C**)—CLSA, (**D**)—CLTA, and (**E**)—CLSTA.

As shown in Figure 7, the model fit well at the initial time steps of the experiment, but its performance declined in the later stages. For an in-depth analysis, this paper presents the scatter plot regression results of the predicted values and actual VO_2_ values of the five models on the test dataset (containing data from three individuals) in Figure 8. The intensity grading thresholds were based on the guidelines proposed by the American College of Sports Medicine (ACSM) in the 11th edition of the ‘Exercise Testing and Prescription Guidelines’: low intensity (<46% VO_2_max, <1.80 L·min^−1^), moderate intensity (46–63% VO_2_max, 1.80–2.47 L·min^−1^), and high intensity (≥64% VO_2_max, ≥2.51 L·min^−1^) [17]. At low intensities, all five models showed a tendency to overestimate, with LSTM showing the largest deviation. As the intensity reached moderate levels, the model prediction shifted to a slight underestimation. Convolutional feature extraction effectively converged the error, with the CNN-LSTM slope approaching 1 and the CLSA points being the most concentrated. During high-intensity exercise, all models showed underestimated prediction errors that were significantly larger than those in the moderate-to-low intensity stages, indicating that the models had difficulty accurately capturing VO_2_ changes in this intensity range. Among them, LSTM performed the worst, while CLSA and CLSTA, which fused channel attention, were closest to the ideal line. The performance of the five models was consistent with the *R*^2^ ranking in Table 4 and Table 5, indicating that ‘convolutional feature extraction + spatial attention’ is an effective means of suppressing errors and improving *R*^2^.

## 4. Discussion

Previous studies predicting VO_2_ during exercise were limited in terms of both ‘input dimensions’ and ‘model depth.’ Most studies only used heart rate, or simply added breathing parameters on top of that; on the other hand, algorithms mainly relied on simple regression or RNN models without the systematic exploration of feature extraction. Lu et al. (2024) used a backpropagation neural network with chest strap ECG/PPG-HR combined with respiratory rate and minute ventilation as input variables to obtain an MAE of 165 mL·min^−1^ [18]. Bangaru et al. (2025) used forearm IMU + electromyography signals and a Bi-LSTM to achieve a lower error of 1.26 mL·kg^−1^·min^−1^, but this required additional sensor deployment [19]. To address the above limitations, this study constructed and compared five models—LSTM, CNN-LSTM, CLSA, CLTA, and CLSTA—within the same dataset. These models combined spatial, temporal, and spatio-temporal attention mechanisms and were evaluated for their performance in predicting oxygen consumption during exercise. Compared with previous studies, we attempted to use commonly available and easily collected three-axis accelerometers and heart rate as dynamic input variables. At the algorithm level, we not only introduced a convolution module to extract local motion patterns but also systematically examined the benefits and limitations of the attention mechanism. The CLSA model with the best performance in the model constructed in this study achieved an *R*^2^ of 0.96, which was an improvement over previous studies. The results showed the following. (1) Combining accelerometer and heart rate data improved the accuracy of oxygen uptake prediction compared to using the heart rate alone. (2) The introduction of the CNN module improved model performance compared to using the LSTM model alone. (3) The introduction of attention mechanisms led to performance fluctuations. Among them, the SAM could improve model performance while the TAM alone did not improve model performance compared to the baseline CNN-LSTM, indicating that attention mechanisms do not always bring gains. At the same time, the CLSTA model, which simply stacked spatial and temporal attention mechanisms, also did not perform optimally. (4) In terms of the predictive accuracy of oxygen uptake at different exercise intensity stages, the five models constructed all showed lower predictive performance at high-oxygen-uptake stages than at moderate and low-oxygen-uptake stages.

### 4.1. Enhanced VO_2_ Prediction Using Accelerometer–Heart Rate Fusion

In this study, we used accelerometer and heart rate signals as dynamic features in the input variables of the VO_2_ prediction model. In fact, accelerometer signals reflect the mechanical work generated by the movement itself while the heart rate reflects the body’s physiological response to the movement stimulus. The two respectively reflect the internal and external load conditions during exercise.

Research indicates that cumulative triaxial acceleration data is highly correlated with various physiological indicators (such as muscle oxygen content and maximum oxygen uptake) [20]. In this study, the fluctuation characteristics of the accelerometer signals (Figure 6A) and their vector integrals VM (Figure 6C) further corroborated the above correlation. As can be seen from Figure 6, VM fluctuated violently during high-intensity exercise and at the beginning of exercise (when the exercise amplitude changed significantly). However, when the subjects adapted to the running rhythm and performed regular exercises with small amplitude changes, the VM fluctuation frequency decreased significantly. This phenomenon was consistent with Sheridan’s limitation that ‘slow movements are easily ignored by the system,’ revealing the bottleneck in identifying low-dynamic activities through accelerometer signals [21].

The heart rate reflects the body’s physiological response to exercise stimuli and is one of the most widely used means of quantifying internal load. This is also consistent with Fick’s principle, whereby an increase in cardiac output increases oxygen delivery and uptake, resulting in a positive correlation between the heart rate and VO_2_ in a steady state [22]. However, using the heart rate alone also has its limitations. On the one hand, the heart rate is influenced by physiological factors such as the maximum heart rate and resting heart rate. On the other hand, in exercises with rapidly changing rhythms, the heart rate alone cannot accurately reflect sudden changes in intensity, and it is easily affected by factors unrelated to exercise (e.g., the heart rate may increase due to emotional tension) [23].

VO_2_ is an output of complex physiological processes and is determined by both external exercise power and internal physiological status. Accelerometer data ensures that the model knows ‘what exercise was performed,’ while heart rate data lets the model know ‘what kind of response the body experienced.’

Previous studies have shown that inputting motion measurement signals such as acceleration and physiological signals such as the heart rate into a non-linear model can significantly reduce VO_2_ estimation errors [21]. As shown in Table 4, this study also found that the combined model was able to capture changes in exercise intensity, thus far exceeding single data source models in terms of prediction accuracy and reliability [24].

### 4.2. The Key Role of CNN in Predicting Oxygen Uptake

In one-dimensional time series applications, CNNs slide over continuous inputs (such as the heart rate or accelerometer signals). This type of local feature learning is well suited for capturing waveform patterns in motion data. This study shows that, compared with the independent LSTM model, introducing a CNN layer can significantly reduce prediction errors and improve the goodness of fit (Table 4). This finding is highly consistent with research conclusions in exercise physiology and related fields. For example, Lee’s energy expenditure study based on IMU found that when predicting steady-state energy expenditure, their CNN-LSTM hybrid model demonstrated the best performance among three models (CNN, LSTM, and CNN-LSTM) [25]. Hossain pointed out in energy estimation research that CNN-LSTM models show better performance than simpler networks [26]. Amelard also found that convolutional networks achieved high VO_2_ prediction accuracy [27]. The CNN layer effectively captures key action features related to VO_2_ changes by extracting local spatial patterns in time series, thereby improving feature extraction capabilities. The subsequent LSTM layers further model the dynamic evolution of these features over time. The combination of the two achieves collaborative modeling of spatial and temporal characteristics, thereby significantly improving the model’s ability to characterize VO_2_ change trends [28].

### 4.3. The Impact of the Attention Mechanism on Predicting Oxygen Uptake

As shown in Table 5, compared with the original CNN-LSTM model without attention mechanisms, the introduction of spatial and temporal attention mechanisms resulted in different changes in the model, indicating that the introduction of attention modules does not necessarily improve performance in all cases. Improper or mismatched attention mechanism designs may cause fluctuations in model performance. This phenomenon is consistent with the conclusions of some existing studies: as pointed out by Vaswani, the use of attention mechanisms needs to be combined with task characteristics for targeted design to avoid blindly adding them and causing negative effects [29]. The CLSTA model, which combines temporal and spatial attention, performs worse than the CLSA model, which only includes spatial attention, on the training set. This suggests that simply stacking temporal and spatial attention modules may introduce optimization conflicts or learning redundancy, thereby weakening the actual improvement of the model. Previous studies have also observed similar phenomena: excessive stacking of attention layers does not effectively fuse multiple dependent features [30,31].

The spatial attention mechanism used in this study employs an SE attention module, which is essentially a channel attention mechanism. In the context of the heart rate and acceleration fusion, different channels represent different physiological meanings: the heart rate channel reflects the heart’s oxygen supply response, while the three acceleration channels reflect the intensity of movement in different directions of the body. The SE attention module can automatically adjust the weights of these channels based on the motion state, allowing the model to focus on more informative signals at different stages [32]. For example, during steady moderate-intensity exercise, the heart rate is approximately linearly correlated with VO_2_ and responds relatively smoothly. At this time, heart rate signals are more indicative of VO_2_ predictions, and the SE module may increase the weight of heart-rate-related features. Similarly, during high-intensity interval training, acceleration signals fluctuate dramatically while the heart rate increases with a delay. The model can use channel attention to focus more on features related to instantaneous exercise intensity in the acceleration channel.

In contrast, in time series applications, the temporal attention mechanism is typically viewed as attention to time steps, i.e., assigning weights to the features at each time point. Ideally, temporal attention allows the model to ‘focus’ on the moments that contribute most to the current VO_2_ prediction [33]. However, physiological changes in the VO_2_ are smooth and continuous, with a delayed effect. When the intensity of exercise changes, oxygen consumption does not instantly reach a new level but gradually changes through several stages. This means that the VO_2_ value at a given moment is the result of the cumulative effect of exercise intensity over a period of time, rather than being determined solely by the current instantaneous heart rate and exercise conditions [34]. After introducing temporal attention, the model may tend to assign excessive weight to certain time points and ignore information from other time periods. For example, the model shown in Figure 6 may overemphasize the heart rate and acceleration peaks at the end of the input sequence, where changes are dramatic. However, due to the lagging characteristics of VO_2_, this approach may mislead the model: short-term dramatic changes do not mean that VO_2_ will immediately surge proportionally. The improper allocation of time attention may sever the continuous cumulative relationship of the VO_2_ signal, causing the model to miss early information that contributes to the current VO_2_.

The results indicate that attention mechanisms have great potential for improving model performance, but their effectiveness depends on reasonable module design and integration methods. In subsequent studies, attention modules will be optimized according to task requirements to maximize performance gains and avoid unnecessary performance degradation.

### 4.4. Increased Error in VO_2_ Prediction Model During High-Intensity Exercise Phases

During high-intensity exercise, all models showed significantly increased prediction errors compared to the moderate- and low-intensity phases. In Figure 8, the deviation of the scatter points from the ideal line was significantly larger in each sub-figure. Amelard also pointed out that deep learning models perform well in VO_2_ time series prediction, but their performance under different exercise intensity conditions needs further validation [27]. First, this may be related to the fact that as exercise intensity increases, physiological responses (such as the relationship between the heart rate and oxygen consumption) become more complex and non-linear. Some literature indicates that at very low or very high exercise intensities, the relationship between the heart rate and VO_2_ becomes significantly non-linear [9]. Secondly, the duration of high-intensity exercise maintained by the subjects was short, resulting in a sample size that was significantly lower than that in the moderate-to-low intensity range. In addition, the attention mechanism had a defect of ‘weak local perception,’ i.e., limited ability to capture instantaneous rapid changes [35]. Finally, during high-intensity exercise (exceeding the lactate threshold or critical power), human VO_2_ kinetics exhibit greater delays and fluctuations [36]. These multiple factors together exacerbated the uncertainty of the model’s predictions during high-intensity exercise phases.

Furthermore, research has shown that when continuous targets have a skewed distribution, the lack of observations in certain intervals makes it difficult for a model to ‘see’ and learn the correct mapping relationships in these intervals, thereby reducing its generalization ability across the entire target range [37]. For the oxygen uptake prediction dataset, high-intensity exercise samples account for only about 36%, which is a relatively small proportion. This imbalance in the target output distribution weakens the model’s generalization performance in the high-intensity range. During training, the model primarily optimizes the overall loss, thus paying more attention to medium- and low-intensity samples, which account for a large proportion of the data, and not paying enough attention to high-intensity samples, which account for a relatively small proportion of the data. In addition, high-intensity exercise data itself may have high physiological heterogeneity and noise. Different individuals have large differences in VO_2_ responses at extreme intensities, making it more difficult to learn reliable patterns when there are insufficient samples. Therefore, for the model used in this study, the high-intensity portion of the training data was relatively limited, causing the model to make predictions based on limited experience in this range, which naturally led to a decrease in accuracy. In future research, we will further consider appropriate data augmentation, reweighting, or stratified modeling for high-intensity samples to mitigate the impact of sample imbalance on the model.

## 5. Conclusions

This study used dynamic and static physiological data obtained from resting test and CPET to construct five models based on LSTM, CNN-LSTM, and CNN-LSTM with three attention mechanisms introduced. The models successfully predicted oxygen uptake during exercise. We proposed two innovations: first, we established a multi-source input fusion strategy to optimize feature representation by combining accelerometer dynamic signals with static heart rate data; second, we designed an attention-optimized path to systematically explore the synergistic mechanisms of three attention mechanisms in the CNN-LSTM architecture. The results indicate the following. (1) Combining accelerometer and heart rate data improves the accuracy of oxygen uptake prediction compared to using the heart rate alone. (2) The introduction of the CNN module is beneficial for improving the performance of the oxygen consumption prediction model. (3) Attention mechanisms do not always improve oxygen uptake predictions, and simply stacking attention mechanisms in a prediction model does not necessarily yield the best results. (4) The model’s predictive performance is poor at high oxygen uptake levels, and further consideration is needed to resolve this issue. This paper not only provides a new methodological reference for predicting physiological parameters but also offers practical application value for real-time monitoring in the field of sports science. However, this study was still limited by its small sample size and limited data diversity. In future studies, we will expand the cross-group sample size and develop high-intensity error compensation algorithms to achieve more accurate oxygen uptake predictions.

## Figures and Tables

**Table 1:** Basic information of the subjects.

	Male (*n* = 14)	Female (*n* = 7)
Age	24 ± 3	25 ± 3
Height (cm)	176 ± 8	163 ± 5
Weight (kg)	70.6 ± 13.3	52.3 ± 6
BMI	22.8 ± 3	19.8 ± 1.5
Body fat percentage (%)	16.2 ± 5.8	23.7 ± 3.4

**Table 3:** Model structure.

	Dynamic Feature Input Layer	Static Feature Input Layer	CNN	SAM	TAM	LSTM	Output Layer
Number of neurons	10 × 5	6	64	64	64	128	1
Activation functions	\	\	ReLU	Sigmoid	Sigmoid	Tanh	Linear

**Table 4:** Performance comparison of feature combinations and models for VO_2_ prediction.

Feature	Model	Train			Validation			Test		
		RMSE	MAE	*R* ^2^	RMSE	MAE	*R* ^2^	RMSE	MAE	*R* ^2^
HR + Static Features	LSTM	0.0851	0.0626	0.9918	0.2006	0.1342	0.9536	0.3335	0.2305	0.8882
	CNN-LSTM	0.0306	0.0224	0.9981	0.2095	0.1477	0.9499	0.3232	0.2950	0.8950
HR + Acc Data + Static Features	LSTM	0.0892	0.0649	0.9908	0.1031	0.0764	0.9871	0.2720	0.2078	0.9256
	CNN-LSTM	0.0044	0.0035	1.0000	0.0504	0.0302	0.9971	0.2317	0.1566	0.9460

**Table 5:** Comparison of VO_2_ prediction performance metrics across models on training, validation, and test sets.

Model	Train			Validation			Test		
	RMSE	MAE	*R* ^2^	RMSE	MAE	*R* ^2^	RMSE	MAE	*R* ^2^
CLSA	0.005	0.0038	1.0000	0.0517	0.0290	0.9968	0.1942	0.1241	0.9621
CLTA	0.0051	0.0040	1.0000	0.0519	0.0285	0.9968	0.2648	0.1881	0.9295
CLSTA	0.0041	0.0031	1.0000	0.0609	0.0304	0.9955	0.2030	0.1279	0.9586

## Data Availability

The data presented in this study are available on request from the corresponding author.

## Abbreviations

The following abbreviations have been used in this manuscript:

VO_2_	Oxygen Uptake
HR	Heart Rate
ACC	Accelerate
CPET	Cardiopulmonary exercise testing
BMI	Body Mass Index
LSTM	Long Short-Term Memory
CNN	Convolutional Neural Network
SAM	Spatial Attention Module
TAM	Time Attention Mechanism
STAM	Spatio-temporal Attention Module
CLSA	CNN-SAM-LSTM model
CLTA	CNN-TAM-LSTM model
CLSTA	CNN-STAM-LSTM model
RMSE	Root Mean Square Error
MAE	Mean Absolute Error

## References

[1] Laukkanen J.A., Isiozor N.M., Kunutsor S.K. (2022). Objectively Assessed Cardiorespiratory Fitness and All-Cause Mortality Risk. Mayo Clin. Proc..

[2] Jones A.M., Carter H. (2000). The Effect of Endurance Training on Parameters of Aerobic Fitness. Sports Med..

[3] Whipp J.B., Ward A.S. (1992). Gas Exchange Dynamics and the Tolerance to Muscular Exercise: Effects of Fitness and Training. Ann. Physiol. Anthropol..

[4] Guazzi M., Adams V., Conraads V., Halle M., Mezzani A., Vanhees L., Arena R., Fletcher G.F., Forman D.E., Kitzman D.W. (2012). Clinical Recommendations for Cardiopulmonary Exercise Testing Data Assessment in Specific Patient Populations. Circulation.

[5] Crouter S.E., Antczak A., Hudak J.R., DellaValle D.M., Haas J.D. (2006). Accuracy and Reliability of the ParvoMedics TrueOne 2400 and MedGraphics VO2000 Metabolic Systems. Eur. J. Appl. Physiol..

[6] Van Hooren B., Souren T., Bongers B.C. (2024). Accuracy of Respiratory Gas Variables, Substrate, and Energy Use from 15 CPET Systems During Simulated and Human Exercise. Scand. J. Med. Sci. Sports.

[7] Wicks J.R., Oldridge N.B., Nielsen L.K., Vickers C.E. (2011). HR Index—A Simple Method for the Prediction of Oxygen Uptake. Med. Sci. Sports Exerc..

[8] Keytel L., Goedecke J., Noakes T., Hiiloskorpi H., Laukkanen R., Van Der Merwe L., Lambert E. (2005). Prediction of Energy Expenditure from Heart Rate Monitoring During Submaximal Exercise. J. Sports Sci..

[9] Davidson P., Trinh H., Vekki S., Müller P. (2023). Surrogate Modelling for Oxygen Uptake Prediction Using LSTM Neural Network. Sensors.

[10] Li F., Chang C.-H., Chung Y.-C., Wu H.-J., Kan N.-W., ChangChien W.-S., Ho C.-S., Huang C.-C. (2021). Development and Validation of 3 Min Incremental Step-In-Place Test for Predicting Maximal Oxygen Uptake in Home Settings: A Submaximal Exercise Study to Assess Cardiorespiratory Fitness. Int. J. Environ. Res. Public Health.

[11] DiPietro R., Hager G.D. (2020). Deep learning: RNNs and LSTM. Handbook of Medical Image Computing and Computer Assisted Intervention.

[12] Porszasz J., Casaburi R., Somfay A., Woodhouse L.J., Whipp B.J. (2003). A Treadmill Ramp Protocol Using Simultaneous Changes in Speed and Grade. Med. Sci. Sports Exerc..

[13] Mei P., Li M., Zhang Q., Li G., Song L. (2022). Prediction Model of Drinking Water Source Quality with Potential Industrial-Agricultural Pollution Based on CNN-GRU-Attention. J. Hydrol..

[14] Guo M.-H., Xu T.-X., Liu J.-J., Liu Z.-N., Jiang P.-T., Mu T.-J., Zhang S.-H., Martin R.R., Cheng M.-M., Hu S.-M. (2022). Attention Mechanisms in Computer Vision: A Survey. Comput. Vis. Media.

[15] Hu J., Shen L., Sun G. Squeeze-and-Excitation Networks. Proceedings of the IEEE Conference on Computer Vision and Pattern Recognition.

[16] Woo S., Park J., Lee J.-Y., Kweon I.S., Ferrari V., Hebert M., Sminchisescu C., Weiss Y. (2018). CBAM: Convolutional Block Attention Module. Computer Vision—ECCV 2018.

[17] American College of Sports Medicine (2021). ACSM’s Guidelines for Exercise Testing and Prescription.

[18] Lu Z., Yang J., Tao K., Li X., Xu H., Qiu J. (2024). Combined Impact of Heart Rate Sensor Placements with Respiratory Rate and Minute Ventilation on Oxygen Uptake Prediction. Sensors.

[19] Bangaru S.S., Wang C., Aghazadeh F., Muley S., Willoughby S. (2025). Oxygen Uptake Prediction for Timely Construction Worker Fatigue Monitoring Through Wearable Sensing Data Fusion. Sensors.

[20] Gómez-Carmona C.D., Bastida-Castillo A., Ibáñez S.J., Pino-Ortega J. (2020). Accelerometry as a Method for External Workload Monitoring in Invasion Team Sports. A Systematic Review. PLoS ONE.

[21] Sheridan D., Jaspers A., Viet Cuong D., Op De Beéck T., Moyna N.M., de Beukelaar T.T., Roantree M. (2025). Estimating Oxygen Uptake in Simulated Team Sports Using Machine Learning Models and Wearable Sensor Data: A Pilot Study. PLoS ONE.

[22] Nakamura T., Kiyono K., Wendt H., Abry P., Yamamoto Y. (2016). Multiscale Analysis of Intensive Longitudinal Biomedical Signals and Its Clinical Applications. Proc. IEEE.

[23] Ernst G. (2017). Heart-Rate Variability—More than Heart Beats?. Front. Public Health.

[24] De Brabandere A., Op De Beéck T., Schütte K.H., Meert W., Vanwanseele B., Davis J. (2018). Data Fusion of Body-Worn Accelerometers and Heart Rate to Predict VO2max during Submaximal Running. PLoS ONE.

[25] Lee C.J., Lee J.K. (2024). IMU-Based Energy Expenditure Estimation for Various Walking Conditions Using a Hybrid CNN–LSTM Model. Sensors.

[26] Hossain M.B., LaMunion S.R., Crouter S.E., Melanson E.L., Sazonov E. (2024). A CNN Model for Physical Activity Recognition and Energy Expenditure Estimation from an Eyeglass-Mounted Wearable Sensor. Sensors.

[27] Amelard R., Hedge E.T., Hughson R.L. (2021). Temporal Convolutional Networks Predict Dynamic Oxygen Uptake Response from Wearable Sensors Across Exercise Intensities. NPJ Digit. Med..

[28] Zhu C., Liu Q., Meng W., Ai Q., Xie S.Q. (2021). An Attention-Based CNN-LSTM Model with Limb Synergy for Joint Angles Prediction. Proceedings of the 2021 IEEE/ASME International Conference on Advanced Intelligent Mechatronics (AIM).

[29] Vaswani A., Shazeer N., Parmar N., Uszkoreit J., Jones L., Gomez A.N., Kaiser Ł., Polosukhin I. (2017). Attention Is All You Need. Advances in Neural Information Processing Systems.

[30] Cao F., Yang S., Chen Z., Liu Y., Cui L. (2024). Ister: Inverted Seasonal-Trend Decomposition Transformer for Explainable Multivariate Time Series Forecasting. arXiv.

[31] Zhou X., Sheil B., Suryasentana S., Shi P. (2024). Multi-Fidelity Fusion for Soil Classification via LSTM and Multi-Head Self-Attention CNN Model. Adv. Eng. Inform..

[32] Zheng B., Luo W., Zhang M., Jin H. (2025). Arrhythmia Classification Based on Multi-Input Convolutional Neural Network with Attention Mechanism. PLoS ONE.

[33] Khan M., Hossni Y. (2025). A Comparative Analysis of LSTM Models Aided with Attention and Squeeze and Excitation Blocks for Activity Recognition. Sci. Rep..

[34] Schneider D.A., Wing A.N., Morris N.R. (2002). Oxygen Uptake and Heart Rate Kinetics During Heavy Exercise: A Comparison Between Arm Cranking and Leg Cycling. Eur. J. Appl. Physiol..

[35] Zhao B., Xing H., Wang X., Song F., Xiao Z. (2023). Rethinking Attention Mechanism in Time Series Classification. Inf. Sci..

[36] Gløersen Ø., Colosio A.L., Boone J., Dysthe D.K., Malthe-Sørenssen A., Capelli C., Pogliaghi S. (2022). Modeling Vo_2_ On-Kinetics Based on Intensity-Dependent Delayed Adjustment and Loss of Efficiency (DALE). J. Appl. Physiol..

[37] Yang Y., Zha K., Chen Y., Wang H., Katabi D. Delving into Deep Imbalanced Regression. Proceedings of the International Conference on Machine Learning, PMLR, Online.

